# Multidrug-Resistant *Salmonella* Typhimurium, Pacific Northwest, United States

**DOI:** 10.3201/eid1310.070536

**Published:** 2007-10

**Authors:** Margaret A. Davis, Thomas E. Besser, Kaye Eckmann, J. Kathryn MacDonald, Donna Green, Dale D. Hancock, Katherine N.K. Baker, Lorin D. Warnick, Yesim Soyer, Martin Wiedmann, Douglas R. Call

**Affiliations:** * Washington State University, Pullman, Washington, USA; † Washington State Department of Health Public Health Laboratories, Shoreline, Washington, USA; ‡ Cornell University, Ithaca, New York, USA

**Keywords:** *Salmonella enterica* serovar Typhimurium, pulsed-field gel electrophoresis, cattle, surveillance, communicable disease, dispatch

## Abstract

We compared human and bovine isolates of *Salmonella enterica* using antimicrobial-drug resistance profiles and pulsed-field gel electrophoresis. From 2000 through 2006, we observed an increase in a novel multidrug-resistant clone of *S*. Typhimurium with no recognized phage type. This clone may represent an emerging epidemic strain in the Pacific Northwest.

Nontyphoidal salmonellosis has been characterized by a pattern of dissemination of clonal *Salmonella enterica*. The most well-known example of this was *S. enterica* serovar Typhimurium definitive phage type 104 (DT104), a clone that emerged and disseminated globally in the 1990s (*1*). Other examples of epidemic clones include *Salmonella* serovar Wien, which spread from North Africa through Europe in the 1970s; *S.* Typhimurium phage type 10 (DT10), which disseminated across Canada in the 1970s; and other phage types of *S.* Typhimurium associated with cattle that disseminated widely in Europe during the 1980s (*2*). More recently, multidrug-resistant (MDR) *S.* Agona was disseminated in poultry in Belgium; MDR *S.* Paratyphi B dT+ in Germany, Belgium, and the Netherlands; and MDR *S.* Newport that harbors the plasmid-mediated AmpC resistance in the United States (*3*). Epidemic clones may remain largely restricted to the animal reservoir for years before their incidence rises among human infections: *S.* Typhimurium phage type 204c disseminated throughout the United Kingdom in cattle from 1980 through 1984, but its proportion among human *S.* Typhimurium infections was not high during that period (*4*). Some epidemic clones, for example DT10 and DT104, expanded in the absence of a specific resistance advantage (*1*). The mechanisms underlying emergence, dissemination, and subsequent decline of novel *Salmonella* clones are unknown, although some researchers have hypothesized that fitness-associated genetic factors may allow more efficient dissemination in specific hosts and environments (*5*) and that acquisition of these fitness genes may be phage mediated (*6*).

One approach to understanding clonal replacement events is to conduct prospective surveillance of *Salmonella* infections in animal and human hosts. We initiated such a study to compare human- and animal-origin *Salmonella* strains by serovar, antimicrobial drug resistance pattern, and pulsed-field gel electrophoresis (PFGE).

## The Study

In Washington, all clinical laboratories and healthcare providers are required to submit *Salmonella* isolates to the Washington State Department of Health Public Health Laboratory (PHL) for serotyping and surveillance (Washington Administrative Code 246–101–201). The PHL is a participant in PulseNet, a national surveillance system established in 1996 that allows state and local public health epidemiologists to compare PFGE profiles of *Salmonella* strains from all US regions (*7*). All human isolates are compared at the PHL by using a standard PFGE PulseNet protocol (*8*). Beginning in January 2004, all human-origin isolates were obtained from the PHL by the Washington State University Zoonosis Research Unit in Pullman. Animal-origin isolates were obtained from the Washington Animal Disease Diagnostic Laboratory in Pullman, the Washington Avian Health Laboratory in Puyallup, and a private veterinary diagnostic laboratory. In addition to isolates originating from clinical veterinary submissions, we obtained isolates from a research study targeting *Salmonella* occurrence on dairy farms in Washington (D. Hancock et al., unpub. data). All isolates were tested for resistance to a panel of antimicrobial drugs with a standard disk-diffusion method (*9*) according to Clinical and Laboratory Standards Institute guidelines (*10*) (Table 1). Isolates collected from January 1, 2000, through April 2005 were tested for susceptibility to a panel of antimicrobial drugs (Table 1). Animal-origin isolates that matched human-origin isolates by serotype and resistance pattern were further compared by using a standard PFGE protocol (*8*). Cluster analysis using the unweighted pair group method with arithmetic mean analysis (UPGMA) of Dice similarity coefficients based on PFGE banding patterns was computed in Bionumerics (Applied Maths, Sint-Martens-Latem, Belgium). To limit the effect of nonindependence among isolates, only the first in each serotype and resistance pattern group within each herd and calendar year was included in the analysis. Statistical significance testing of the change in proportions was conducted in EpiInfo version 6.0 with χ^2^ for linear trend (*11*).

**Table 1:** Trends in resistance of bovine-origin *Salmonella S.* Typhimurium PFGE types TYP035 and TYP187*

Resistance characteristics†	2001	2002	2003	2004	2005	2006
TYP035						
ASu			1	1	1	
ACaz						1
AKSuT		1			1	
AKSSuT	7	7	1	4	1	4
ACKSSuT	1	1				
AKSSuTCaz				3		
AKSxtSSuT				1		
ACKSSuTCaz			2	2		
ACKSxtSSuT					1	
AKSuTCaz					1	
AKSSuTCaz					1	1
ACKSSuTCaz					2	1
ACKSxtSSuTCaz		1	1	6	4	
AGKSxtSSuTCaz						1
Total	8	10	5	17	12	8
TYP187						
ACSSuTCaz				1		
AKSSuTCaz					1	12
AKSuTCaz					1	
Total				1	2	12

*PFGE, pulsed-field gel electrophoresis. †A, ampicillin; Su, triple-sulfa; Caz, ceftazidime; K, kanamycin; T, tetracycline; S, streptomycin; C, chloramphenicol; Sxt, trimethoprim-sulfamethoxazole; G, gentamicin. In April 2005 nitrofurantoin, trimethoprim, and ciprofloxacin were dropped from the panel, and amoxicillin-clavulanic acid, nalidixic acid, and ceftazidime-clavulanic acid were added.

We observed an increase in bovine-origin *S.* Typhimurium isolates that were represented by 2 highly similar PFGE patterns, identified as Washington State PulseNet types TYP035 and TYP187 (Table 2). They were clearly distinguishable from phage type DT104 strains isolated during the same time frame (Appendix Figure). To determine whether these *Xba*I PFGE types were clonal, we characterized 60 *S.* Typhimurium isolates, including 32 TYP035-TYP187 isolates from bovines and 19 TYP035-TYP187 isolates from humans, by PFGE following digestion of DNA by *Spe*I. *Spe*I patterns within each *Xba*I type showed only minor banding variations. Thirteen isolates that were *Xba*I-TYP035 had *Spe*I patterns indistinguishable from those of *Xba*I-TYP187 isolates. In the cluster analysis of *Spe*I patterns, the TYP035 and TYP187 isolates’ *Xba*I patterns were at least 90.1% similar compared to an overall 83.7% similarity for all *S.* Typhimurium isolates in the analysis. Characterization of isolates within the *Xba*I PFGE type TYP035 also included plasmid profiles of 38 isolates with published plasmid-profiling methods (*12*). Isolates were selected for plasmid profiling to maximize the diversity of resistance patterns, geographic origin, and year of isolation. Plasmid sizes were estimated relative to a BAC-Tracker Supercoiled DNA ladder (EPICENTRE Biotechnologies, Madison, WI, USA). Fifteen of the 38 tested isolates harbored a single ≈120-kb plasmid. The remainder had variable plasmid profiles, but the plasmid profiling data did not correlate with resistance phenotype or *Spe*I-banding variations (data not shown). Similar results were obtained with phage typing of a convenience sample of 31 isolates by Rafiq Ahmed at the National Microbiology Laboratory, Winnipeg, Manitoba, Canada. All 31 TYP35 isolates were phage-type untypable with the standard Colindale panel of phages, although an additional set of phages were able to discriminate among some of them (R. Ahmed, pers. commun.). The resulting categories of untypable phages (UT2, UT5, UT7, and UT8) were also not consistent with either plasmid profile or resistance phenotypes (data not shown).

**Table 2:** Human- and bovine-source *Salmonella* Typhimurium isolates from Washington that were *Xba*I-PFGE type TYP035 or TYP187*

Year	Human				Bovine		
	TYP035	TYP187	Total no.† (%)‡		TYP035	TYP187	Total no.† (%)‡
2000	1	0	222 (0.5)		16	0	61 (26.2)
2001	1	0	174 (0.6)		18	0	47 (38.3)
2002	8	0	143 (5.6)		10	0	31 (32.3)
2003	13	0	153 (8.5)		5	0	13 (38.5)
2004	7	0	143 (4.9)		10	1	26 (42.3)
2005	18	0	124 (8.9)		12	2	29 (48.3)
2006	3	7	118 (8.5)		8	12	39 (51.3)

*For human isolates only a single isolate per individual was included, and for bovine isolates only independent isolates were included. PFGE, pulsed-field gel electrophoresis. †Total number of unique *S.* Typhimurium isolates. ‡Percentage of the total that were part of the TYP035-TYP187 clade.

The proportion of all bovine independent *S.* Typhimurium isolates in the TYP035–TYP187 clade increased significantly from 2000 to 2006 in Washington (χ^2^ for linear trend, p<0.01). The proportion also increased significantly among human *S.* Typhimurium isolates during the same period (χ^2^ for linear trend, p<0.01) (Table 2). Expansion of TYP035 apparently occurred in the bovine population of the Pacific Northwest before its increase among human isolates because in 2000 it represented over one quarter of all bovine *S.* Typhimurium, but it was uncommon among human-source *S.* Typhimurium before 2002 to 2003 (Table 1). Similarly, TYP187 was detected in bovine isolates 2 years before its detection in human isolates. Resistance characteristics varied among TYP035 and TYP187 isolates. Before 2003, the predominant resistance pattern among TYP035 isolates was the AKSSuT phenotype; after 2002, isolates were distributed more widely among several resistance patterns (Table 1). The predominant resistance pattern among isolates of TYP187 was AKSSuTCaz (Table 1).

## Conclusions

The strain type reported here may be an emerging epidemic clone of MDR *Salmonella* with the potential to expand further. Since 2001, TYP035 has been infrequently reported in the National PulseNet Database, and most sporadic cases were from Washington. The TYP187 profile has never been reported outside of Washington. Comparison of PFGE patterns between Northwest and New York State *S.* Typhimurium isolates at the Cornell University Zoonosis Research Unit showed no occurrences of TYP035 or TYP187 among 23 bovine and 29 human *S.* Typhimurium isolates from the same period. These comparisons with isolates from other parts of the United States suggest that the emergence of the TYP035/ TYP187 clade is currently focused in the Pacific Northwest region but has the potential to become more widespread. In 2006, the Massachusetts Department of Public Health investigated an outbreak of salmonellosis associated with owl pellets among elementary school students (46 primary cases, 12 secondary cases) (*13*). This outbreak was caused by TYP035 *S.* Typhimurium, and the supplier of the owl pellets was located in a rural county in south-central Washington. This outbreak illustrates that epidemic clones may disseminate widely by means of unpredictable vehicles and that nonfoodborne transmission can have a major public health impact.

The TYP035/TYP187 clade first increased in the animal reservoir, followed by spillover into the human population, consistent with its being an emergent clone. Whether or not this strain will continue to expand, further characterization of this PFGE type and continued surveillance will be important steps for improving our understanding of epidemic *Salmonella* clones (*3*,*5*).

## Supplementary Material

Appendix Figure Dendogram of cluster analysis of pulsed-field gel electrophoresis (PFGE) banding patterns of XbaI-digested Salmonella enterica serotype Typhimurium DNA. A, ampicillin; C, chloramphenicol; K, kanamycin; Sxt, trimethoprim-sulfa; S, streptomycin; T, tetracycline; Su, sulfonamine; Caz, ceftazidime. TYP004 is the PFGE type characteristic of DT104. Each isolate represented here is a unique bovine-origin isolates (each is from a different herd, year, and resistance type). WA, Washington; ID, Idaho.
