# Supplementary figures and images for: Multidrug-Resistant Salmonella Typhimurium, Pacific Northwest, United States
Source: Emerg Infect Dis. 2007 Oct;13(10):1583–6. doi: 10.3201/eid1310.070536 (PMC2851523; doi:10.3201/eid1310.070536)

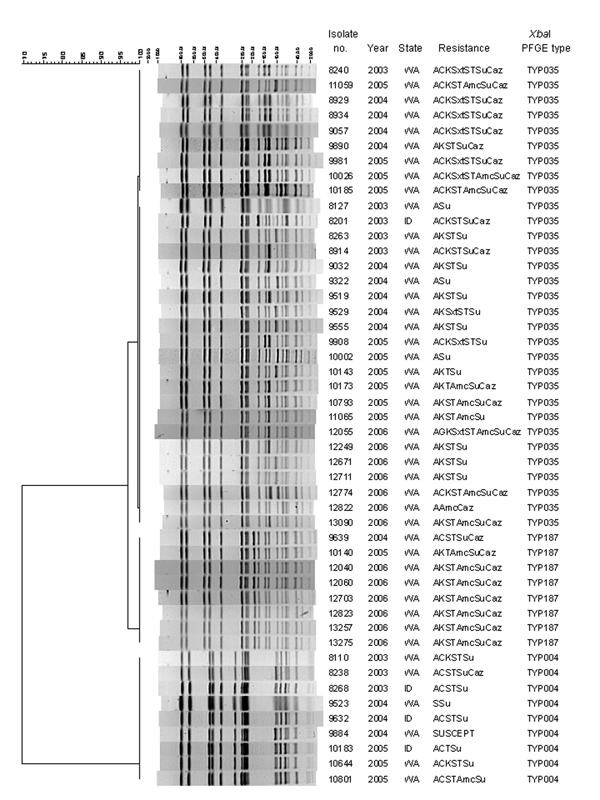

Supplement: Appendix Figure — Dendogram of cluster analysis of pulsed-field gel electrophoresis (PFGE) banding patterns of XbaI-digested Salmonella enterica serotype Typhimurium DNA. A, ampicillin; C, chloramphenicol; K, kanamycin; Sxt, trimethoprim-sulfa; S, streptomycin; T, tetracycline; Su, sulfonamine; Caz, ceftazidime. TYP004 is the PFGE type characteristic of DT104. Each isolate represented here is a unique bovine-origin isolates (each is from a different herd, year, and resistance type). WA, Washington; ID, Idaho. [file 07-0536_appF-s1.gif]
